# Amitriptyline-Induced Hyperprolactinemia in a Pediatric Patient

**DOI:** 10.7759/cureus.59604

**Published:** 2024-05-03

**Authors:** Safia Centner, Felicia Cooper, Shilpa Gurnurkar, Shirin Hasan

**Affiliations:** 1 Medicine, University of Central Florida College of Medicine, Orlando, USA; 2 Pediatric Endocrinology, Nemours Children's Hospital, Orlando, USA; 3 Child and Adolescent Psychiatry, Nemours Children's Hospital, Orlando, USA

**Keywords:** tricyclic antidepressant, pharmacological side effects, hyperprolactinemia, amitriptyline, prolactin

## Abstract

Hyperprolactinemia is an endocrinological disorder that might arise from various physiologic or pathologic conditions, as well as from pharmacologic sources. These pharmacologic sources include antidepressants, antipsychotics, and dopamine receptor-blocking agents. Amitriptyline is classified as a tricyclic antidepressant. While it is FDA-approved primarily for the treatment of depression, amitriptyline also demonstrates efficacy in managing various other conditions, such as anxiety, post-traumatic stress disorder, insomnia, chronic and neuropathic pain, and migraine prevention. We present a case of a 10-year-old patient with a history of autism spectrum disorder (ASD), attention-deficit/hyperactivity disorder (ADHD), and migraine headaches who was incidentally found to have elevated prolactin levels while taking amitriptyline for migraine prophylaxis. While risperidone, an antipsychotic that can be used for ASD management, is commonly known to induce hyperprolactinemia, the association between amitriptyline and elevated prolactin is less frequently described in the literature. This case underscores the necessity for healthcare providers across various specialties to be aware of amitriptyline-induced hyperprolactinemia.

## Introduction

Hyperprolactinemia is an endocrinological disorder that may arise from various physiologic or pathologic conditions, as well as from pharmacologic sources [1]. Hyperprolactinemia is defined as a prolactin level greater than 15 to 20 ng/mL, depending on the laboratory and assay used [1]. Any factor impacting the secretion or elimination of prolactin can influence its levels in the bloodstream [1]. Physiological hyperprolactinemia is more transient and adaptive, whereas pathological and pharmacological hyperprolactinemia is typically symptomatic and may cause enduring, lasting effects [1].

Physiologic causes of hyperprolactinemia include pregnancy, exercise, stress (including anxiety surrounding phlebotomy), sleep, seizures, nipple stimulation, and lactation [1]. Pathologic causes are pituitary or hypothalamic disease and/or tumors, systemic disorders, idiopathic, ectopic production, genetic causes, and medications [1]. Common pharmacologic causes include estrogen therapy, antipsychotic medications (such as risperidone and haloperidol), anticonvulsants, opioids, and dopamine receptor-blocking agents [2]. Other pharmacologic causes are less commonly seen, including tricyclic antidepressants (such as amitriptyline and clomipramine) and selective serotonin receptor inhibitors (such as fluoxetine) [3].

Patients with elevated levels of prolactin may be asymptomatic, or they can present with galactorrhea or hypogonadism [1]. Most symptoms are typically seen in premenopausal women and men, with the hallmark signs of reproductive dysfunction and galactorrhea [1]. Pediatric patients may also present with pubertal delay and amenorrhea [1]. Other signs to monitor include menstrual cycle abnormalities, decreased bone mass, decreased libido, headaches, or visual field defects [1].

If a patient is showing the symptoms above, a serum prolactin level is recommended [1]. The ideal time to draw this lab is during the midmorning hours and with the patient fasting, though this is not essential [1]. Once hyperprolactinemia is found, an extensive history and physical examination are important to consider or exclude potential causes [1]. Laboratory tests, such as thyroid hormone levels, luteinizing hormone, follicle-stimulating hormone, and testosterone/estradiol (depending on gender), should be ordered, as well as an MRI of the pituitary gland [1]. 

Management depends on the exact cause of the abnormal prolactin, the level of elevation, and the degree of symptoms. Treatment options include surgery, dopamine agonists, and/or radiation [1]. For drug-induced hyperprolactinemia, the medication should be discontinued, if feasible, to determine if the prolactin level normalizes [1].

## Case presentation

A 10-year-old male with a medical history of autism spectrum disorder (ASD), attention-deficit/hyperactivity disorder (ADHD), developmental delay, and migraines presented to our endocrinology clinic for consultation on increased prolactin levels.

**Figure 1:**
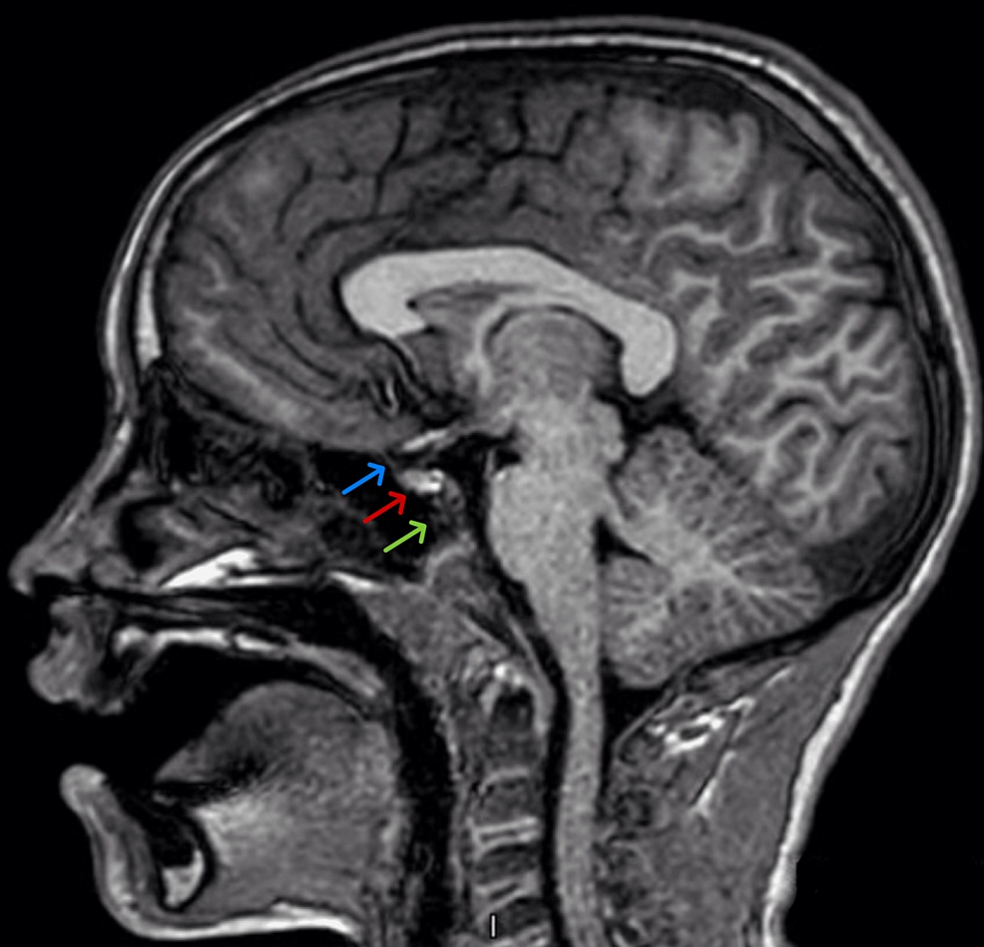
Sagittal T1 Brain MRI Magnetic resonance imaging shows no brain pathology and a normal pituitary stalk (blue arrow), anterior pituitary (red arrow), and posterior pituitary (green arrow).

The patient was following up with a pediatric neurologist for his migraines and had been taking amitriptyline for migraine prophylaxis for the previous year. Besides the amitriptyline, he took rizatriptan as needed for migraine abortive treatment. He was also under the care of a psychiatrist for his ASD and ADHD and for the management of aggression, mood lability, hyperactivity, impulsivity, and self-injurious behaviors. He was started on a trial of guanfacine, which was helpful with his hyperactivity but seemed to have no effect on the aggression. It was suggested that the patient begin risperidone, and baseline labs were ordered. These labs were drawn when the patient was under sedation for an MRI of the brain obtained for further evaluation of the migraines. The results showed an elevated prolactin level of 50.6 ng/mL (reference range for males in Tanner stage 1: ≤ 10 ng/mL), as shown in Table 1. At this time, he was referred to the endocrinology department for further evaluation prior to starting risperidone. Non-contrast MRI did not show any brain pathology, and the visualized pituitary gland was normal-appearing, as shown in Figure 1.

**Table 1:** Patient’s prolactin levels (ng/ml) before and after amitriptyline discontinuation This table shows the patient’s original elevated prolactin level of 50.6 ng/mL and the next measured prolactin level of 4.37 ng/mL following amitriptyline discontinuation. The reference range of ≤ 10 ng/mL for the prolactin level refers to males in Tanner Stage 1.

	Prolactin level (ng/mL)	Reference range (ng/mL)
Before amitriptyline discontinuation	50.6	≤ 10
After amitriptyline discontinuation	4.37	≤ 10

The patient was then evaluated in the endocrinology clinic. He denied galactorrhea and had no abnormal findings on a physical examination. He was at Tanner stage I for puberty, at a chronological age of 10. Amitriptyline was suspected to be the etiology of the hyperprolactinemia, and therefore it was suggested that the medication be held (if that was a viable option for the patient) and labs repeated. The patient’s neurologist agreed, and the amitriptyline was discontinued. The patient was placed on propranolol for migraine prophylaxis instead. Two months later, the labs were repeated under sedation (due to severe needle phobia). The prolactin level had normalized to 4.37 ng/mL, as shown in Table 1. The patient’s migraines were reported to be under good control on propranolol. He started risperidone a few weeks later, as prescribed by his psychiatrist for behavioral concerns.

## Discussion

This case describes a patient with elevated levels of serum prolactin, likely caused by amitriptyline. The patient was sedated during the blood draw, decreasing the suspicion of anxiety at the time of lab collection as the cause of hyperprolactinemia. Furthermore, he had no other risk factors for hyperprolactinemia, and an MRI of the brain revealed a normal-appearing pituitary gland. The suspicion of amitriptyline as the cause of hyperprolactinemia was confirmed after a repeat laboratory draw showed normal serum prolactin after discontinuation of the amitriptyline.

Risperidone, an antipsychotic medication that is used for ASD management, is associated with a 30% increased risk of hyperprolactinemia [4,5]. This medication acts as an antagonist at dopamine D2 receptors but is also an agonist at serotonin receptors like 5HT2A [4]. Dopamine acts as a key regulator of prolactin secretion by inhibiting the release of prolactin from the pituitary gland. When the action of dopamine is blocked or reduced, prolactin secretion can increase, leading to hyperprolactinemia.

Hyperprolactinemia can also be caused by amitriptyline, albeit less frequently [3,6]. Amitriptyline is a tricyclic antidepressant that exerts its therapeutic effects primarily by inhibiting the reuptake of serotonin and norepinephrine in the brain [7]. The increased levels of these neurotransmitters in the synaptic cleft enhance neurotransmission and alleviate symptoms of depression [3]. The medication has been FDA-approved to treat depression but has been known to be helpful to patients with anxiety, insomnia, post-traumatic stress disorder, chronic pain, and migraines [7]. Migraines are related to the vasodilation of cranial vessels and are associated with low serotonin levels [8]. Therefore, the increase in serotonin associated with amitriptyline use helps alleviate migraine symptoms.

**Figure 2:**
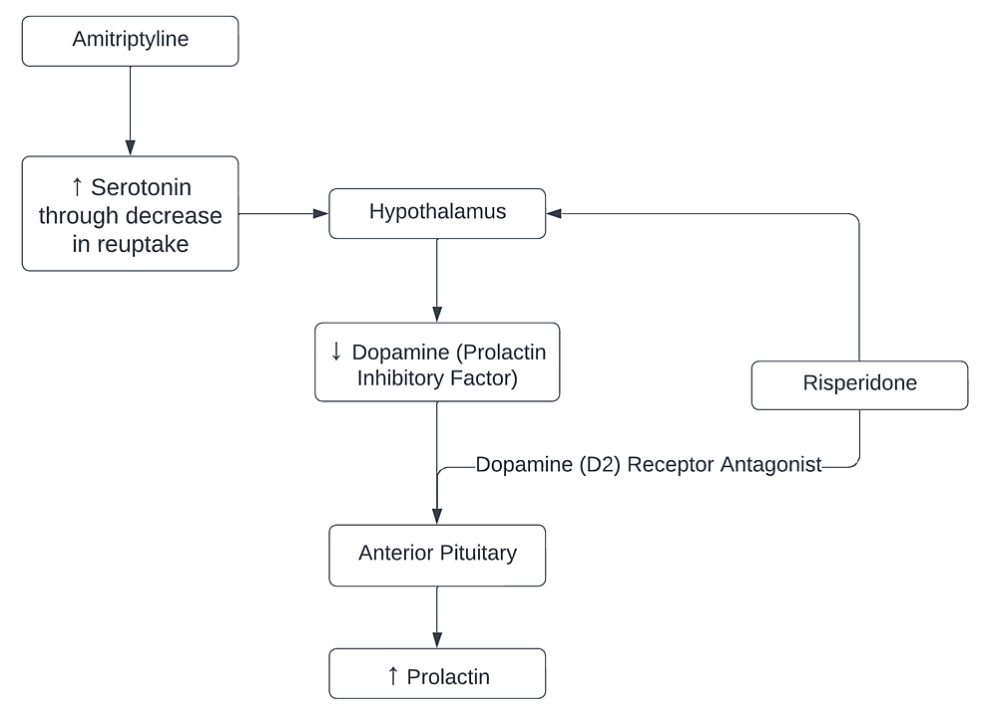
Effect of amitriptyline and risperidone on prolactin secretion Risperidone is a dopamine (D2) receptor antagonist and decreases the synthesis of dopamine, resulting in hyperprolactinemia. Amitriptyline leads to an increase in serotonin, resulting in decreased dopamine and a resultant increase in prolactin. The figure has been created by the authors.

Common side effects of amitriptyline include weight gain, gastrointestinal issues, headaches, and drowsiness, with an FDA warning for suicidal ideation and behavior in adolescents and young adults [4]. However, the effect on serotonin and norepinephrine may also indirectly decrease dopamine activity in certain regions, thus reducing dopamine’s inhibitory effect on prolactin release [7]. While amitriptyline's impact on dopamine receptors is generally less pronounced compared to medications such as risperidone (which directly antagonizes dopamine receptors), it can potentially interfere with dopamine's inhibitory effect on prolactin release [9]. Consequently, the disruption in the balance of these neurotransmitters can contribute to elevated prolactin levels. This pathway is demonstrated in Figure 2. Furthermore, amitriptyline possesses anticholinergic properties [7]. Acetylcholine is also involved in the regulation of prolactin secretion, and by blocking its action, amitriptyline may indirectly lead to increased prolactin levels [10].

## Conclusions

We report a case of amitriptyline-induced hyperprolactinemia in a pediatric patient taking this medication for migraine prophylaxis. As with any medication, it is essential for patients and healthcare providers to be aware of potential side effects and to monitor them during treatment. Clinicians should remain vigilant for rare but significant side effects, such as hyperprolactinemia, when prescribing amitriptyline, particularly in pediatric patients. This case exemplifies the importance of coordinated care among specialties. Timely recognition and interdisciplinary collaboration between pediatricians, neurologists, psychiatrists, and endocrinologists are crucial for optimizing patient outcomes and preventing potential complications when patients are on psychotropic medications.
